# AI-powered 3D pathology protocol enhances enteric nervous system visualization and quantification for clinical diagnostics

**DOI:** 10.7150/thno.112024

**Published:** 2025-06-20

**Authors:** Young Hyun Yun, Kee Young Chung, Yunjoo Lee, Tae Sik Sung, Dayoung Ko, Seung-Bum Ryoo, Hyun-Young Kim, Kyu Joo Park, Jong Pil Im, Byeong Gwan Kim, Joo Sung Kim, Seong-Joon Koh, Hyung Jin Choi

**Affiliations:** 1 Department of Anatomy and Cell Biology, Seoul National University College of Medicine, Seoul, Republic of Korea.; 2 Department of Biomedical Sciences, Seoul National University College of Medicine, Seoul, Republic of Korea.; 3 Ragon Institute of Massachusetts General Hospital, Massachusetts Institute of Technology, and Harvard, Cambridge, Massachusetts, United States.; 4 Department of Surgery, Seoul National University College of Medicine, Seoul, Republic of Korea.; 5 Department of Pediatric Surgery, Seoul National University Children's Hospital, Seoul, Republic of Korea.; 6 Department of Internal Medicine and Liver Research Institute, Seoul National University Hospital, Seoul, Republic of Korea.; 7 Department of Pediatric Surgery, Seoul National University College of Medicine, Seoul, Republic of Korea.; 8 Neuroscience Research Institute, Seoul National University College of Medicine, Seoul, Republic of Korea.; 9 Wide River Institute of Immunology, Seoul National University, Seoul, Republic of Korea.; 10 Department of Brain and Cognitive Sciences, Seoul National University, Seoul, Republic of Korea.

**Keywords:** enteric nervous system, tissue clearing, artificial intelligence, ulcerative colitis, Hirschsprung disease

## Abstract

**Rationale:** Accurate diagnosis and understanding of gastrointestinal (GI) diseases such as ulcerative colitis and Hirschsprung's disease remain challenging due to the limitations of traditional two-dimensional (2D) histopathology in capturing the intricate three-dimensional (3D) architecture and dynamic microenvironment of GI tissues. This study explores the potential of integrating 3D imaging techniques with artificial intelligence (AI)-based analysis to improve histological evaluation and diagnostic accuracy.

**Methods:** Using advanced imaging and computational tools, we identified critical structural and functional details of the enteric nervous system and associated tissues that are often missed by 2D approaches.

**Results:** The results showed that 3D imaging coupled with AI significantly improves diagnostic accuracy and provides new insights into disease mechanisms, enabling earlier and more precise detection of pathological changes. In addition, this approach enhances our understanding of the pathophysiology of GI diseases, bridging gaps in both clinical and basic research.

**Conclusions:** These findings underscore the transformative potential of 3D imaging and AI to revolutionize diagnostic workflows and advance our knowledge of GI diseases, ultimately contributing to improved patient outcomes and innovative research methodologies.

## Introduction

The gastrointestinal (GI) system contains an autonomous neural network, known as the enteric nervous system (ENS), responsible for regulating various GI functions. The ENS is comprised of plexuses, ganglia, and motor neurons, where the myenteric and submucosal plexuses are the essential constituents. The ENS, extending from the esophagus to the anal sphincter and even influencing organs such as the liver and pancreas, plays a vital role in the regulation of GI functions 1. Nevertheless, disturbances in the ENS can lead to enteric neuropathies, including Hirschsprung disease (HSCR), a congenital disorder caused by the absence of ganglion cells in the distal colon, leading to impaired motility and functional obstruction. This condition leads to severe constipation, abdominal distension, and, in some cases, life-threatening enterocolitis 2. Prompt diagnosis and timely intervention are crucial for managing HSCR effectively 3. Accumulating evidence indicates a correlation between the ENS and other GI diseases including colorectal cancer and inflammatory bowel disease (IBD), highlighting its possible importance in the progression of such diseases 4, 5.

For decades, conventional two-dimensional (2D) pathology has been the cornerstone for diagnosing and understanding GI diseases 6. Efforts are ongoing to utilize artificial intelligence (AI) in these traditional histopathologic assessments to enhance clinical metrics 7. However, limitations in 2D histopathology highlight its inability to fully capture the structural complexity and three-dimensional (3D) context of GI tissues, including various cell types, extracellular matrices, and vasculature 8. This led to a study in which optical tissue clearing of the mouse intestine was performed to observe the myenteric plexus (MP), small nerve fibers, and epithelium by fluorescent antibody staining 9. Additionally, 2D pathology cannot provide insight into dynamic GI tissue microenvironments, such as cellular interactions and immune responses within complex three-dimensional neuron structures. This inadequacy results in incomplete understanding of disease mechanisms and sampling bias 10. Practical constraints also limit the number of tissue sections analyzed from a specimen, potentially overlooking crucial regions or variations containing essential diagnostic information. The preparation process for 2D histology, involving fixation, sectioning, and staining, can introduce artifacts that compromise diagnostic accuracy 11. These limitations hinder comprehensive visualization and study of intricate tissue interactions in their natural context 12, 13.

Recent studies highlight the substantial advantages of 3D imaging in analyzing tissue structure and disease 8. Unlike traditional 2D methods, which offer limited depth and obscure spatial relationships, 3D histology techniques like tissue clearing and volumetric imaging have revolutionized understanding of complex tissues. These methods have demonstrated potential for comprehensive structural analysis in human lungs, liver, lymph nodes, and brain disease tissues, including Alzheimer's and Parkinson's diseases 14-17. Although the critical roles of the ENS in GI and central nervous system (CNS)-related disorders, the ENS remains less well-characterized than the CNS, particularly with respect to image-based quantitative analysis. Consequently, there has been an increase in the number of efforts aimed at refining the structural mapping of the ENS through the use of whole-mount tissue processing and 3D imaging approaches. Given the utility of 3D imaging and its advantages in disease-related research, this technique has also been applied to the human ENS 18. Recently, full-thickness samples of the sigmoid colon were rendered transparent without sectioning, followed by whole-mount immunolabeling and 3D imaging. This approach enabled detailed 3D graphical rendering of the ENS architecture within the muscularis externa, and allowed for comparative analysis of signal intensities for various neuronal markers localized to that layer 19. A novel 3D approach for characterizing intestinal mucosal structures in IBD, particularly in ulcerative colitis (UC), Crohn's disease (CD), and non-IBD cases, introduced a standardized method to quantify structural differences between normal and IBD-associated lesions 20. Furthermore, a 3D method analyzing human ENS anatomy without tissue sectioning revealed significant differences in ENS features between HSCR colons and pediatric or adult donor tissues, highlighting age-related changes and unique features in the HSCR transition zone 12, 21.

Combined with 3D imaging, AI-powered image analysis significantly enhances tissue analysis accuracy and efficiency 22. AI software employs machine learning algorithms requiring user training for data analysis and pattern recognition, enabling custom optimizations for specific research purposes 23, 24. In contrast, non-AI software is immediately usable, focusing on image processing and 3D visualization 25. AI-powered technology has clear advantages over non-AI methods in image analysis and pattern recognition, offering greater accuracy and efficiency. Previous studies have demonstrated that AI-powered methods excel in pathology 26, yet despite these advances, AI-powered 3D imaging has not yet been applied to the study of the ENS, highlighting an area that remains unexplored in current research.

The objective of this study was to investigate the efficacy of tissue clearing, antibody staining, 3D imaging, and AI-powered analysis in facilitating rapid and accurate diagnoses. We hypothesized that AI-powered 3D imaging and analysis would significantly enhance diagnostic efficiency, accuracy, and reproducibility compared to traditional methods, enabling faster processing, improved visualization of crypts and neural structures, and more precise quantitative analysis for GI diseases. Additionally, we set out to compare the diagnostic accuracy and efficiency of non-AI and AI-powered visualization and analysis in human colon biopsy and surgical samples. Moreover, it was hypothesized that the presentation of quantitative metrics from colon biopsy and surgical samples would facilitate the evaluation of histological structures and enhance diagnostic precision.

## Methods

### Tissue acquisition

Colon biopsy specimen from a patient diagnosed with UC (IRB No. 2012-115-1183) and one from a healthy control subject (IRB No. 2011-202-1178) were obtained via colonoscopy with approval from the Seoul National Hospital of Institutional Review Board. Human surgical colon specimens, including those from HSCR (IRB No. 2320-072-1475), were acquired with the authorization of the Institutional Review Board for Human Subjects Research at Seoul National University Hospital.

### Subjects

Tissue samples were obtained from three subjects: a patient with UC, a healthy control, and a patient diagnosed with HSCR. The UC patient was a 34-year-old male with severe disease activity, as indicated by a Mayo Endoscopic Subscore of 3 at the time of sampling. The healthy control subject was a 19-year-old male with no endoscopic abnormalities. The HSCR patient was a 1-month-old male infant who presented with delayed passage of meconium after birth. A rectal biopsy confirmed the absence of ganglion cells, establishing the diagnosis of HSCR.

### Tissue processing and clearing

A colon biopsy tissue obtained from endoscopy was fixed overnight in 4% paraformaldehyde (PFA) at 4 °C. After washing in 1x phosphate-buffered saline (PBS) (30 min on a shaker at room temperature), the tissue was transferred to a decolorization solution (5% CHAPS, 12.5% N-Methyldiethanolamine in PBS) at 37 °C for 1 day to remove residual blood. The tissue was then washed in PBS (30 min on a shaker at room temperature) and then placed in an electrophoretic tissue device (Binaree, Cat No. BDTC-003) with Rapid Clearing solution (Binaree, Cat No. BRTC-001), applying 1.5 mA at 35 °C for 4 hours to facilitate rapid lipid removal. The cleared tissue was washed in PBS (30 min at room temperature), incubated in electrophoretic tissue clearing (ETC) solution (4 hours on a shaker at 55 °C) to eliminate remaining lipids, and washed in PBS (30 min at room temperature). The timeline and procedure comparison for colon biopsy sample processing show that both 2D and 3D immunohistochemistry (IHC) methods take approximately one week. However, 2D IHC requires 2.5 days for embedding and sectioning, plus one day for imaging, while 3D IHC minimizes manual labor and captures hundreds of high-resolution images in just 30 minutes.

Surgical colon tissues were immediately resected into 1×1 cm^2^ and pinned with margins using insect pins. The tissues were fixed in 4% PFA at 4 °C for 2 days, washed in PBS (1 x 3 hours on a shaker at room temperature), and placed in an ETC chamber (Logos, Cat No. C30001). With 4% electrophoretic tissue clearing solution circulated (Logos, Cat No. C13001), 1.5 mA was applied across the tissue at 35 °C for 16 hours to effectively clear lipids and other optical barriers. The cleared tissue was washed in PBS (1 x 3 hours at room temperature), incubated in ETC solution (8 hours on a shaker at 55 °C) to further remove residual lipids, and washed in PBS (1 x 3 hours on a shaker at room temperature). For surgical samples, the 2D and 3D IHC protocols each required 12 days from tissue processing to imaging. However, the 3D IHC technique enabled high-resolution imaging in just one day, while the 2D approach required 3 days for embedding and sectioning, followed by at least 3 days for imaging.

### Tissue immunostaining

Colon biopsy and surgical tissues were processed as follows: Both types of tissues were incubated with primary antibodies diluted in DeepLabel™ Solution B (Logos, Cat No. C33003) (26 hours for colon biopsy tissue; 2 days and 12 hours for surgical tissues). The primary antibodies included rabbit anti-beta III tubulin (1:500; Abcam, Cat No. ab18207) for both colon biopsy and surgical tissues. Biopsy tissue was additionally incubated with mouse anti-E Cadherin (1:200; Abcam, Cat No. ab76055), while surgical tissues were incubated with mouse anti-NeuN IgG 488 conjugated (1:200; Merck, Cat No. MAB377X). After washing in PBS (30 min for colon biopsy tissue; 3 hours for surgical tissues), the tissues were incubated with secondary antibodies in DeepLabel™ Solution B: donkey anti-rabbit IgG H&L 488 (1:500; Jackson, Cat No. 711-545-152) and donkey anti-mouse IgG H&L 594 (1:500; Jackson, Cat No. 715-585-150) for colon biopsy tissue, and donkey anti-rabbit IgG H&L 488 (1:500; Jackson, Cat No. 711-545-152) for surgical tissues, for 1 day. All tissues were then incubated in C Match solution (Crayon, Cat No. 50-3010) for 1 day to achieve refractive index (RI) matching. All procedures were performed at 37 °C in the dark with gentle shaking.

### Image acquisition

The colon biopsy tissue was placed on a 35 mm confocal dish and immobilized on a 25 mm glass coverslip. The tissue was then immersed in C Match solution and imaged using a C2+ upright confocal microscope (Nikon Instruments, Yokohama, Japan) with a Plan-Apochromat 10x lens (NA = 0.5, WD = 5.5 mm) at 3 μm intervals. The excitation and long-pass emission filters employed were Alexa Fluor 488 and Alexa Fluor 594. For the surgical tissues, they were placed on a 60 mm confocal dish and immobilized on a 25 mm glass coverslip. These samples were also immersed in C Match solution and imaged using the C2+ upright confocal microscope at 15 μm intervals (15% overlap). Additionally, to visualize large regions of the surgical tissues, a Light-sheet 7 fluorescence microscope (Carl Zeiss, Oberkochen, Germany) with a 5x imaging lens (NA = 0.1, RI = 1.53) and 5x illumination lenses (NA = 0.16, RI = 1.53) at 4 μm intervals (15% overlap) was utilized. For these images, the excitation and long-pass emission filter used was Alexa Fluor 488.

### Image analysis

To facilitate subsequent analysis, the raw images of the surgical samples obtained with the light-sheet system were converted to an image file with a resolution of 30% of the original, as the file size reached 374 GB. This was conducted using ZEN software (version 3.10, Carl Zeiss), as it was a setting that the analysis PC could handle. The acquired images were analyzed using IMARIS software (v10.0.3, Oxford Instruments) and Aivia software (v13.0, Leica Microsystems). These software tools were utilized for visualizing and quantifying crypt maximum and minimum diameters, single crypt length and area, single crypt volume and surface, multiple crypt volume, axon fiber count, neuron volume, axon fiber length and diameter, and MP volume and surface area. All statistical analyses, including the creation of bar graphs and violin plots, were conducted using Microsoft Excel and Prism (v8.0.2, GraphPad Software).

Analysis timelines for colon biopsy samples show that manual 2D methods take at least 13 days to analyze 400 images. In contrast, the 3D analysis method reduces this to one day. A 3D AI-powered method, after a one-day training period, allows rapid analysis in 20 minutes, offering approximately a 1000-fold increase in efficiency. For surgical samples, manual 2D analysis takes 30 days, whereas 3D analysis requires only one day. Following a one-day training, the AI-powered analysis completes the task in just 4 hours, improving efficiency by around 26 times.

### Statistics

Statistical analyses were performed using Prism 8 (GraphPad Software, San Diego, CA). Single crypt volume values were compared among the manual, non-AI, and AI-powered methods using one-way ANOVA followed by Tukey's HSD post hoc test to assess differences.

## Results

### Optimized workflow and timeline for 3D tissue clearing technology and AI-powered imaging analysis

**Figure 1:**
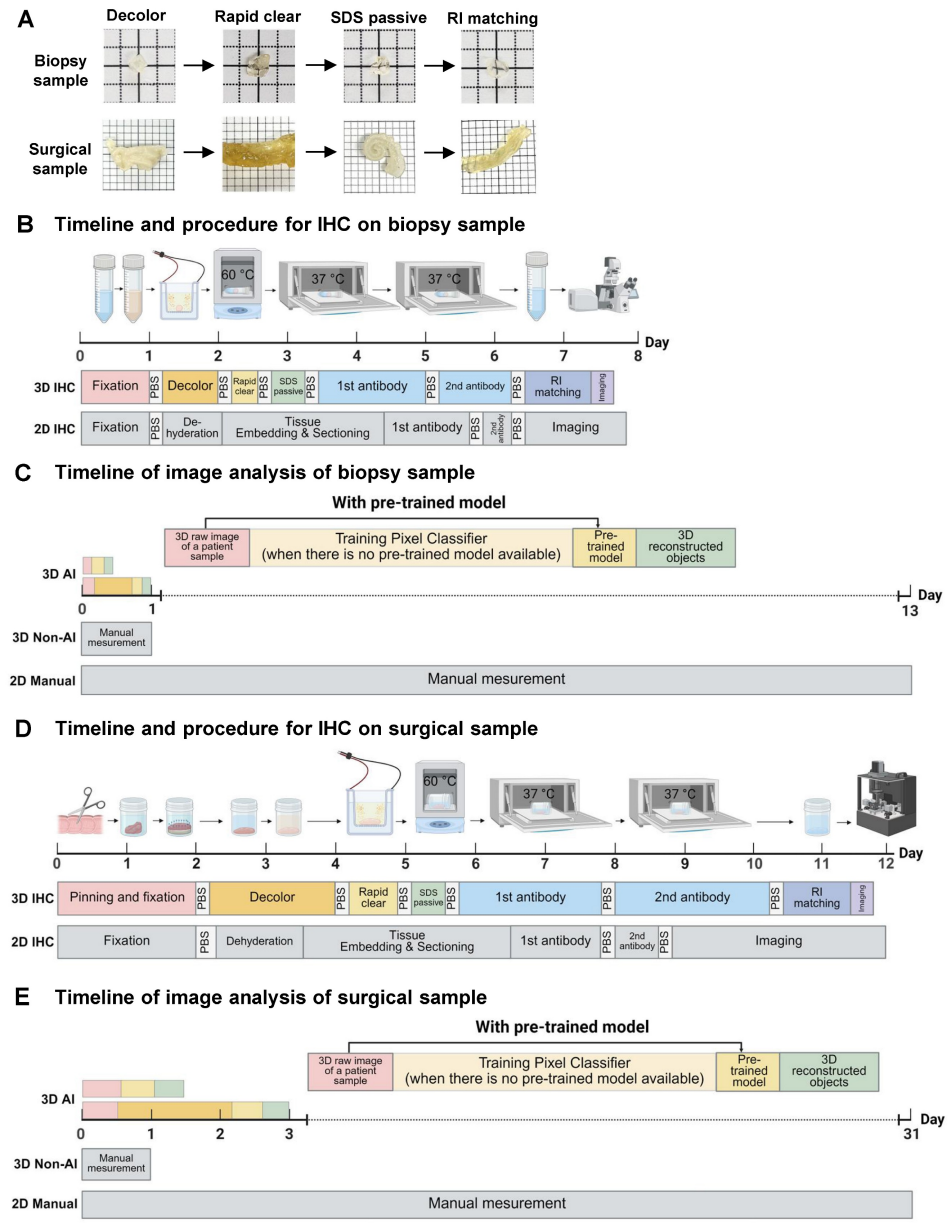
** Colon biopsy and surgical sample processing workflow and non-artificial intelligence (AI) and AI-powered image analysis.** (A) Colon biopsy and surgical samples at various stages of the tissue clearing protocol. (B) Timeline and procedures for two-dimensional (2D) and three-dimensional (3D) immunohistochemistry (IHC) of colon biopsy samples. (C) Timeline for image analysis of colon biopsy samples using manual counting, non-AI, and AI-powered software. (D) Timeline and procedures for 2D and 3D IHC of surgical samples. (E) Timeline for image analysis of surgical samples using manual counting, non-AI, and AI-powered software.

The overall workflow and efficiency improvements of the optimized 3D tissue clearing and AI-powered analysis are summarized in Figure 1A-E. Figure 1A shows the clearing process using decolorization, electroporation, and RI matching to enhance tissue transparency. Figure 1B compares the timelines for 2D and 3D IHC in colon biopsy samples, with 3D imaging completed in 30 minutes. Figure 1C demonstrates that analysis time for biopsy samples is reduced from 13 days using manual 2D methods to just 20 minutes with AI-powered 3D analysis. Figure 1D presents similar processing durations for surgical samples between 2D and 3D IHC, but with faster imaging in 3D. Figure 1E shows that AI-powered 3D analysis shortens surgical sample analysis from 30 days to 4 hours, offering approximately 25.7-fold increase in efficiency.

### Workflow for AI-powered 3D image processing of colon biopsy and surgical colon samples

**Figure 2:**
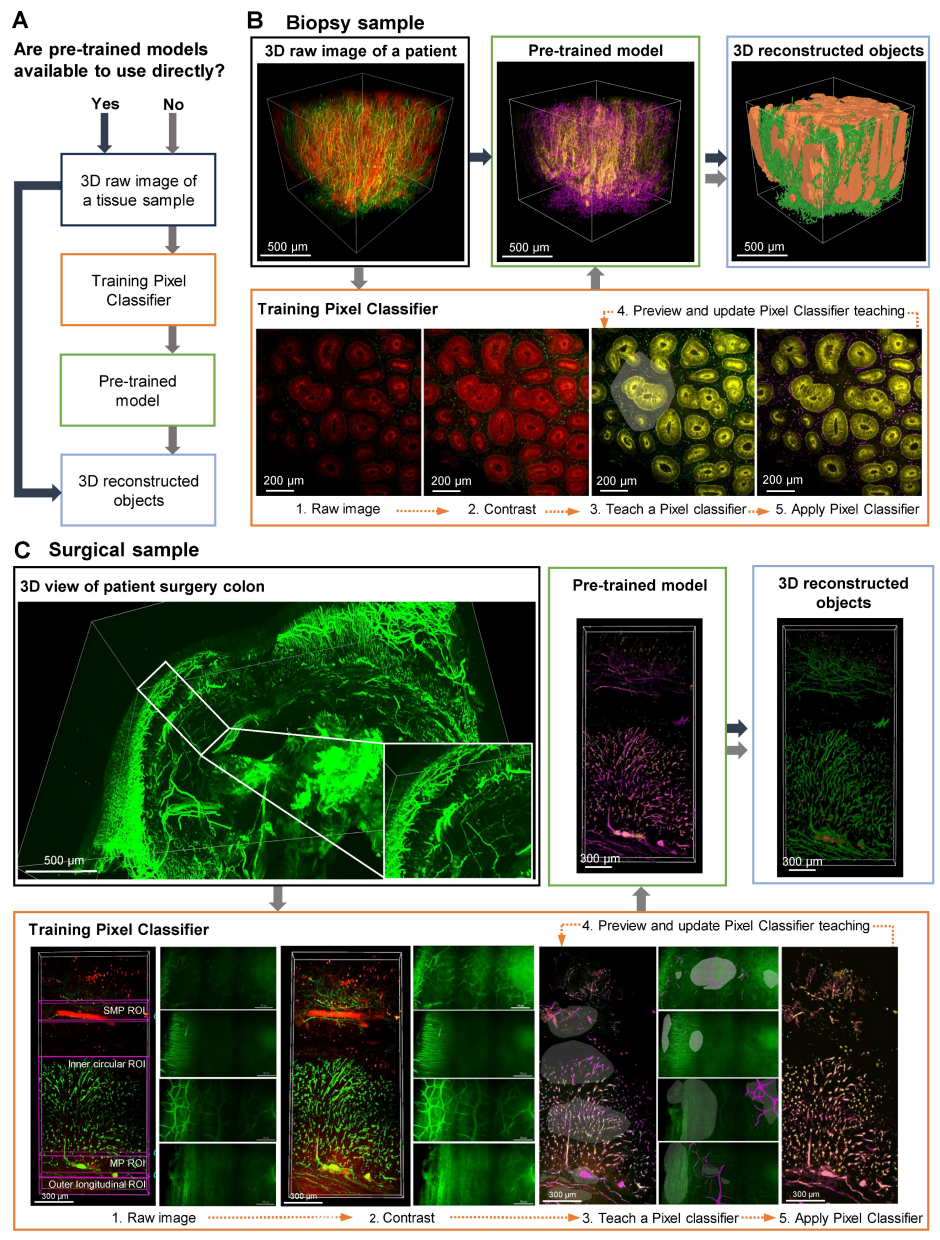
** Artificial intelligence (AI)-powered image analysis workflow for three-dimensional (3D) image processing of mucosal-submucosal colon biopsy and full-thickness surgical colon samples.** (A) AI-powered workflow for a 3D raw image of tissue sample, with and without pre-trained models. (B) Training a pixel classifier on a raw image creates a pre-trained model for a 3D image of colon biopsy sample. (C) Training a structure per plexus layer results in a pre-trained model for a 3D image of a surgical sample.

A detailed, step-by-step description of the AI-powered 3D image analysis pipeline is provided in Supplementary Material 1. Figure 2A presents the workflow for employing AI analysis software to process 3D raw image data obtained from tissue clearing samples. The use of pre-trained models enables the direct conversion of 3D raw images into 3D reconstructed objects in tissue samples. In cases when pre-trained models are not available, training a pixel classifier on the dataset is required; this process involves classifying the raw images before generating the corresponding 3D reconstructed objects.

Figure 2, B and C depict AI-powered 3D image processing of colon biopsy and surgical samples. In these cases, AI training of desired shapes, patterns, and signals is achieved by manually designating fluorescence channels in 2D raw images, followed by training the AI to distinguish between background and signals using the Pixel Classifier tool (Video S1). Preview images are used to evaluate and validate the learned patterns, and upon satisfactory training, a pre-trained model including 3D reconstruction is generated. The final 3D outputs of these AI-segmented models are shown with rotational views in Video S2. For colon biopsy specimens, the AI was trained to identify crypt structures and nerve structures (Figure 2B). Additionally, for surgical specimens, since the full tissue thickness (from mucosa to serosa) can be imaged, the AI was trained on larger colonic structures, including the MP, neural structures in the muscle layer, and the submucosal plexus (SMP) (Figure 2C).

### Comparison of non-AI 3D reconstructed and AI-powered 3D reconstructed colon biopsy colon sample

**Figure 3:**
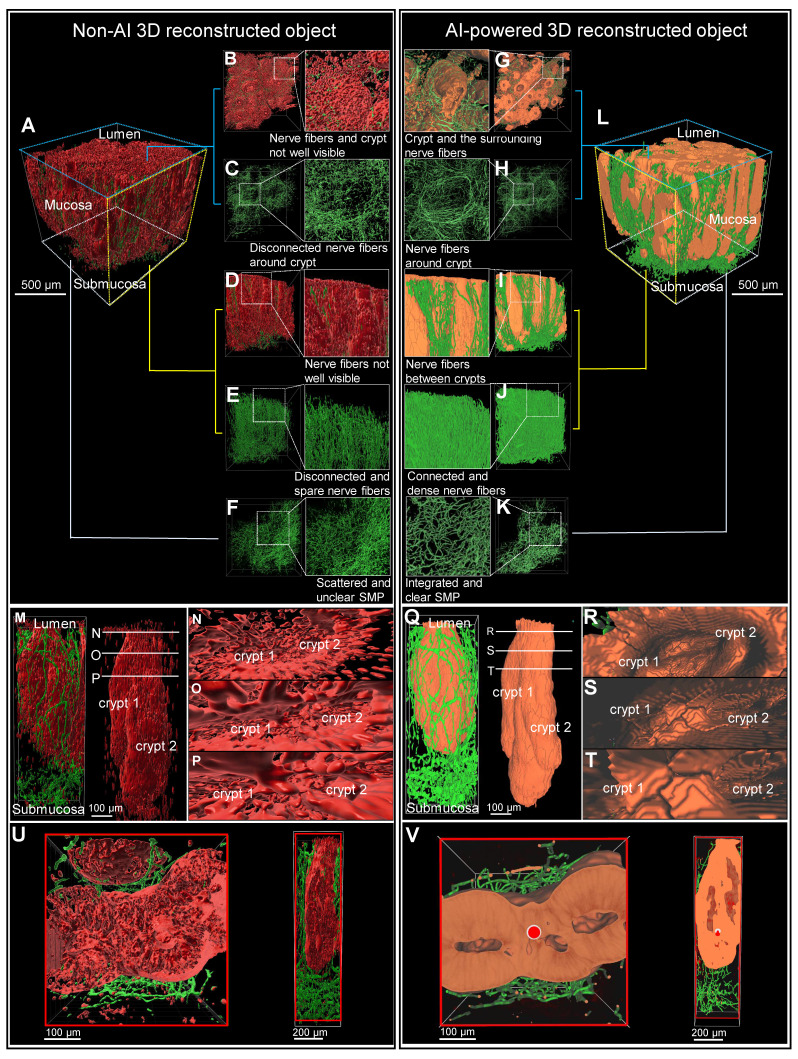
** Comparison of non-artificial intelligence (AI) and AI visualization of a mucosal-submucosal colon biopsy sample.** (A) Three-dimensional (3D) reconstructed non-AI image of the colon biopsy sample showing epithelial cells (red) and nerve fibers (green). (B) Top view and enlarged view of multiple crypt structures from the non-AI image. (C) Top view and enlarged view of a neuron structure in the non-AI image. (D) Side view and enlarged crypt structure in the non-AI image. (E) Side view and enlarged neuron structure in the non-AI image. (F) Bottom view of the neuron structure in the submucosal plexus (SMP) region of the non-AI image. (L) AI-powered image of the colon biopsy sample with epithelial cells (orange) and nerve fibers (green). (G) Top view of crypts and surrounding nerve fibers in the non-AI image. (H) Incomplete AI-powered 3D structure of the crypts (top view). (I) Side view and enlarged crypt structure in the AI-powered image. (J) Side view and enlarged neuron structure in the AI-powered image. (K) Bottom view of the neuron structure in the SMP region in the AI-powered image. (M-O) Internal views of crypt walls. (P) Crypts and surrounding nerve fibers. (Q) Complete AI-powered 3D structure of the crypts. (R-T) Internal views of crypt walls. (U) Cross-section of an incomplete crypt structure in the non-AI 3D image. (V) Cross-section of a fully reconstructed crypt in the AI-powered 3D image.

The 3D image of a colon biopsy sample, stained with the pan-neuronal marker Tuj1 and the crypt structure marker E-Cadherin, was reconstructed using simple fluorescence intensity-based software and compared with results from AI software training (Figure 3, A and L). The AI-powered 3D reconstructed object exhibited enhanced structure and connectivity in depicting crypts (red) and surrounding nerves (green) compared to traditional non-AI 3D reconstruction object (Figure 3, B and G). The AI-powered reconstructions of the mucosal layer's neural structures are notably more connected (Figure 3, C and H). In the side view of the AI image, the nerve fibers passing between the crypts are more visible and the nerves are denser and more connected than in the non-AI 3D reconstruction (Figure 3, D to J). Additionally, AI-trained results showed improved connectivity among neural structures in the submucosal layer (Figure 3F and K). These 3D image results could also be viewed and compared from a variety of angles via video (Video S3).

Magnification of a crypt section revealed that non-AI 3D reconstruction allowed for only rough identification of crypt and neural structures, with clarity diminished by background noise (Figure 3, M to P). In contrast, AI-powered 3D images (Figure 3, Q to T) displayed more interconnected neural structures and a clearly visible distorted crypt structure (Figure 3, Q to T).

In transverse and longitudinal sections of the traditional reconstruction, the anastomosis of crypts due to inflammation-induced distortion was difficult to observe in non-AI reconstructions (Figure 3U). Conversely, the AI-powered 3D reconstruction provided clearer visualization of the complex structures at the center and sides of the crypt, along with surrounding neural structures (Figure 3V).

### Quantitative analysis comparison between manual, non-AI 3D reconstructed and AI-powered 3D reconstructed colon biopsy sample

**Figure 4:**
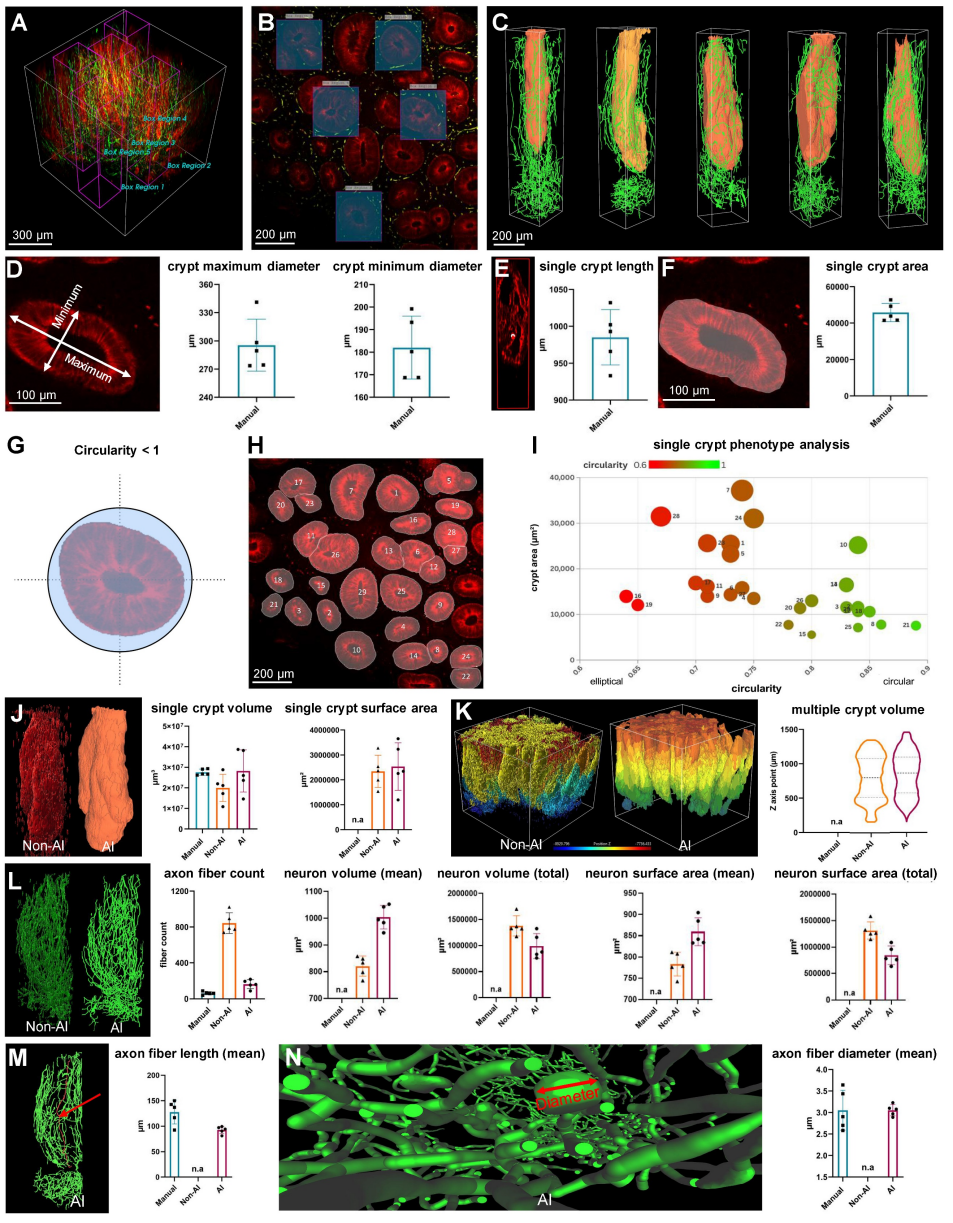
** Comparison between non-artificial intelligence (AI) and AI-powered analysis of a colon biopsy mucosal-submucosa sample.** (A and B) Five crypt region of interest (ROI) regions of a patient colon biopsy sample. (C) Three-dimensional (3D) reconstructed images of ROI regions from patient colon biopsy sample image. (D) A measurement of the maximum and minimum axes in a crypt cross-section. (E) A measurement of the length of the crypt depth. (F) A measurement of the crypt area. (G) Conceptual schematic for crypt circularity. (H) Categorization of crypts by type on a colon biopsy sample two-dimensional image. (I) Single-crypt phenotyping considers the area and circularity of the crypt. (J) A comparison of 3D image analysis methods using manual, non-AI, and AI method. (K) Comparison of 3D images and volume quantitative values between non-AI and AI for multiple crypts. (L) Comparison of 3D images and quantitative metric values between non-AI and AI for crypt neuronal axonal fibers. (M) Comparison of manual and AI-powered length measurements for axon fiber length. (N) Comparison of manual and AI-powered measurements of axon fiber diameter.

The pre-trained model in the image analysis software enabled the semi-automated recognition of neurons in addition to the crypt, as well as the measurement and calculation of both structures. To assess the neural and crypt structures in colon biopsy samples, five regions of interest were randomly selected from the 3D images (Figure 4, A and B). These images were generated using AI-powered 3D reconstruction techniques (Figure 4C).

A manual analysis was conducted to serve as the gold standard for comparison of the performance of AI and non-AI methods. This included the diameter, length, and area of the crypt, and were taken from one 2D image layer of the 3D image (Figure 4, D to F). Additionally, circularity, a measure of structural roundness, was evaluated as a comparative assessment of distorted crypts in lesion tissue. A numeric value of 1 represents a circle, and lower values indicate more atypical and distorted crypts (Figure 4G). After excluding the off-screen crypt in one image layer, distinct IDs were assigned to the remaining recognized crypts (Figure 4H), and phenotypic comparisons were performed on individual crypts using plots that considered circularity and area (Figure 4I). This allowed a relative quantitative assessment of normal and abnormal crypt structure in the lesion.

The neurons surrounding a crypt, rendered as a 3D object, were quantified and compared using the manual, non-AI, and AI methods. For crypt volume, the AI-powered method showed no statistically significant difference from the gold-standard manual method (p > 0.05), indicating close alignment. In contrast, the non-AI method exhibited a statistically significant difference compared to the manual method (p < 0.05), suggesting that the AI-powered method more reliably approximates the gold standard (Figure 4J). There was no significant difference between the AI and non-AI methods for the crypt surface area and total volume of multiple crypt structures within the mucosal area rendered in 3D (Figure 4K). For axon fiber count, the AI-powered method was more similar to the gold standard manual method than the non-AI method due to the inaccurate and numerous three-dimensional object clutter caused by the fluorescence intensity-based 3D reconstruction of the non-AI method (Figure 4L). For neuron volume and neuron surface area, there were significant differences between the AI-powered and non-AI methods (Figure 4L).

For the axon fiber length, there was no significant difference between the gold standard manual method and the AI-powered method. The non-AI method could not quantify the axon fiber length. Since the 3D volume and surface area of neurons cannot be manually measured, and metrics like total neuron path length and average length are also challenging to obtain with non-AI software, AI software was essential for calculating these three-dimensional metrics for neurons.

### Comparison of non-AI 3D reconstructed and AI-powered 3D reconstructed surgical colon sample

**Figure 5:**
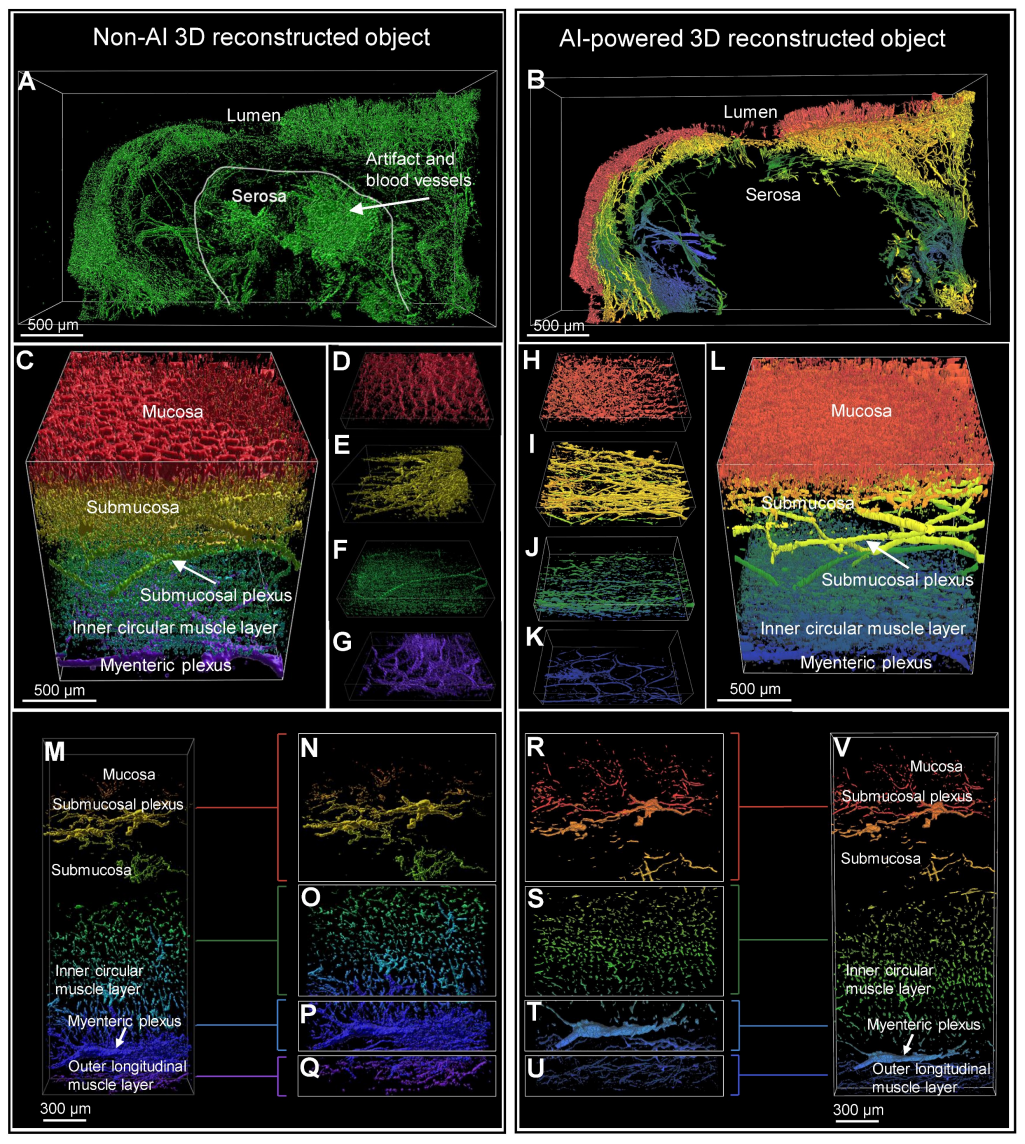
** Comparison of non-artificial intelligence (AI) and AI visualization of surgical full-thickness samples.** (A) Non-AI 3D light sheet image of full-thickness enteric nervous system (ENS) in an Hirschsprung disease (HSCR) patient's surgical sample. (B) AI-enhanced three-dimensional (3D) light sheet image of full-thickness ENS in an HSCR patient sample. (B-V) Both non-AI and AI-generated 3D images of a HSCR surgical sample were color-coded by layer, with red representing the mucosa and blue the serosa. (C-G) Non-AI 3D confocal images of full-thickness ENS in an HSCR sample. (H-K) AI-powered 3D confocal images of full-thickness ENS in an HSCR sample. (M-Q) Non-AI confocal images of an HSCR sample. (R-V) AI-powered 3D confocal images of an HSCR sample.

Figure 5, A and B show the 3D images of a HSCR patient surgical colon sample stained with the pan-neuronal marker Tuj1. These images were captured by light-sheet microscopy and subsequently processed using basic fluorescence intensity-based software (Video S2). The results were then compared with those derived from AI software training. The non-AI method showed autofluorescence from complex and unnecessary vascular structures in the serosal region of the intestinal tissue (Figure 5A). In contrast, the AI-powered method effectively identified not only signal of interest but also morphology and location, allowing for the exclusion of non-specific regions prior to 3D reconstruction (Figure 5B). The difference between the two methods can also be seen in 3D rendering results, as the non-AI method has difficulty separating 3D objects by layer from mucosa to serosa, while the AI-powered method can be trained by layer structure, highlighting and quantifying structural objects in a specific layer. Figure 5, C to G and H to L, captured by confocal microscopy, provide a comprehensive view of the full-thickness connectivity of nerve fibers in the normal region of HSCR patient's surgical colon sample, thereby depicting the neural structures within each individual layer. The SMP and myenteric plexus are clearly shown in the AI-powered reconstructed objects, highlighting the connectivity of the nerves. Figure 5, M to Q and R to V are also confocal images of normal region tissues of HSCR patients, showing detailed neural structures from the mucosal layer to the MP, and especially the MP and muscle neural structures are more clearly visualized with the AI method than in the non-AI method when comparing Figure 5, P and Q and T and U.

### Quantitative analysis comparison between 3D reconstructed and AI-powered 3D reconstructed surgical colon sample

**Figure 6:**
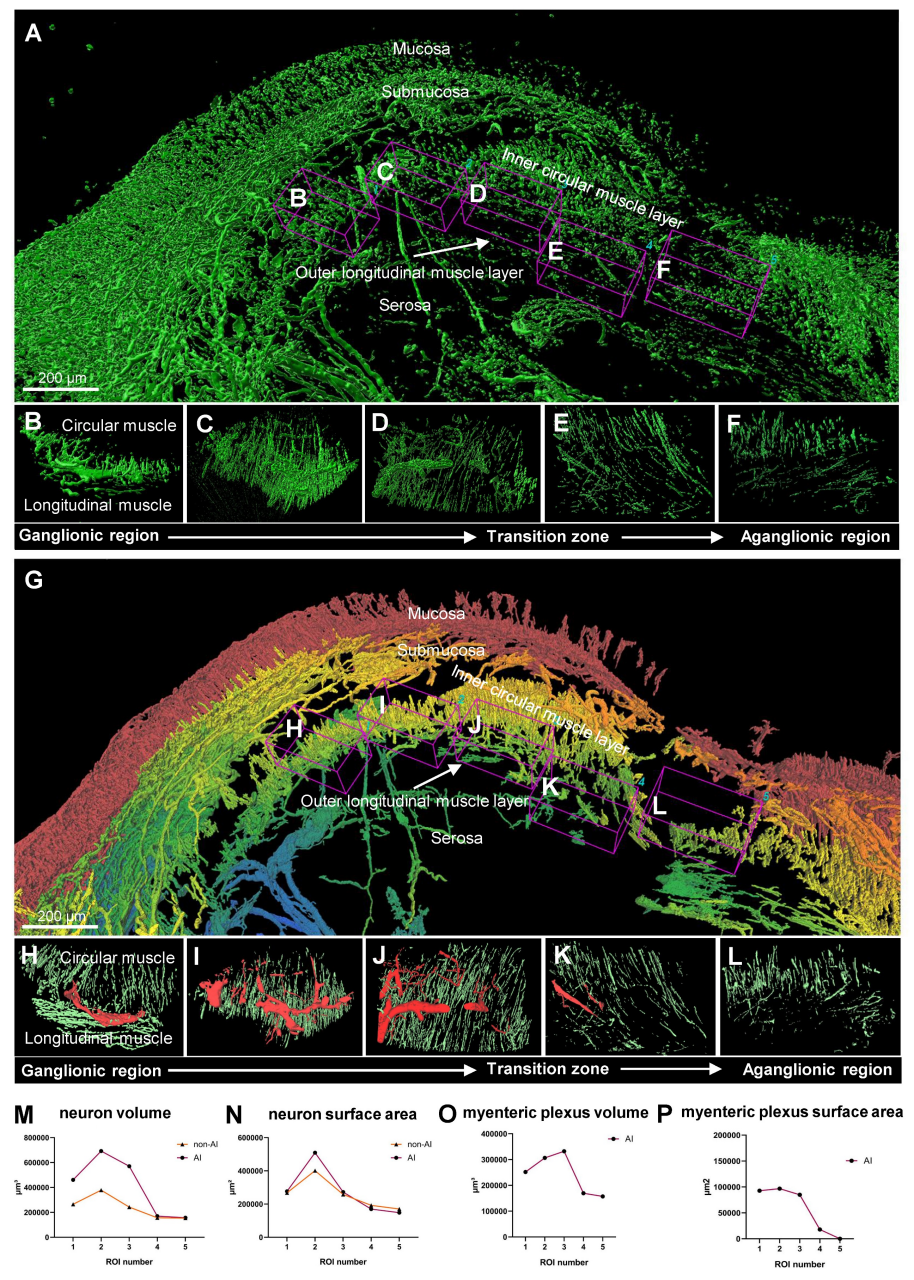
** Comparison between non-artificial intelligence (AI) and AI-powered analysis of patient surgical samples.** (A) Non-AI three-dimensional (3D) image of a Hirschsprung disease (HSCR) patient's surgical sample. (B-D) Non-AI 3D images of ganglionic region in a HSCR sample. (E) Non-AI 3D image of transition zone in HSCR sample. (F) Non-AI 3D image of aganglionic region in HSCR sample. (G) AI-powered 3D image of a HSCR patient's surgical sample with a gradient color coding applied to tissue layers, showing the mucosa in red gradually transitioning to blue at the serosa. (H-J) AI-powered 3D images of ganglionic zone in HSCR sample. (K) AI-powered 3D image of transition zone in HSCR sample. (L) AI-powered 3D image aganglionic region in HSCR sample. (M and N) Quantitative comparison between Non-AI and AI-powered analysis in total enteric nervous system. (O and P) Quantitative analysis of myenteric plexus from AI-powered 3D images.

The comparison between non-AI and AI-powered quantitative analysis of surgical samples from a patient with HSCR is illustrated in Figure 6. Non-AI 3D imaging techniques were utilized to capture the surgical sample, with the ganglionic region detailed in multiple images to highlight the presence of ganglion (Figure 6, A to D). Additionally, the transition zone (Figure 6E) and the aganglionic region (Figure 6F) were depicted using non-AI methods. In contrast, the AI-powered technique provided layer-specific distinction via color coding, which was not achievable with the conventional method. It subsequently generated a 3D image of the same surgical sample, visualizing the ganglionic zone (Figure 6, G to J), as well as the transition and aganglionic regions (Figure 6, K to L). Quantitative comparisons between the metrics of the total ENS obtained through non-AI and AI-powered method analyses are presented (Figure 6, M to N). While the non-AI method yielded a high number of neurons that resulted in similar trends to the AI-powered quantification results, the values were generally higher in most regions. The MP was further examined through quantitative analysis using AI-powered images (Figure 6, O to P), as the non-AI method could not measure the MP alone.

## Discussion

Our study introduces an efficient workflow for colon biopsy and surgical sample processing using AI-powered 3D tissue-clearing technology. Optimized protocols tailored to sample characteristics significantly reduce processing times compared to traditional methods, requiring approximately 8 days for biopsies and 12 days for surgical samples. By automating 3D reconstruction through AI-powered pre-trained models, analysis times were reduced by up to 1000-fold for biopsies and 30-fold for surgical samples. This automation also minimizes human error and enhances reproducibility and reliability.

AI-powered image processing provided superior visualization and quantitative analysis compared to non-AI methods, delivering higher-resolution images with clearer structural details, such as intact crypts and well-connected nerve fibers. These advancements improve the accuracy and efficiency of pathological diagnoses while reducing labor intensity. Additionally, automated quantitative analysis ensures consistency, offering reliable data critical for clinical decision-making and highlighting the transformative potential of AI-powered 3D imaging in pathology.

The AI-powered 3D tissue imaging technology developed in this study offers transformative potential for clinical diagnosis and personalized treatment planning. It enables detailed evaluation of micropathological changes, such as crypt distortion in early IBD or high-risk populations 27, 28, through quantitative 3D analysis. Additionally, it supports precision medicine applications by predicting treatment responses and prognostic outcomes in IBD. AI-powered analysis also enhances the understanding of the ENS in GI diseases, such as its interaction with immune cells in IBD and its role in GI tumors 29, 30. For instance, perineural invasion, a critical prognostic factor in colorectal cancer 31, is challenging to identify with traditional 2D imaging but is clearly discernible using AI-powered 3D imaging.

Beyond IBD and colorectal cancer, this technology addresses unmet needs in other conditions like irritable bowel syndrome (IBS) 32. While IBS diagnosis currently relies on symptom evaluation and exclusion of other conditions, AI-powered 3D imaging can provide deeper insights into structural alterations in the ENS, complementing studies of neural changes observed in the brain. Moreover, advanced techniques, such as histo-cytometry 33, or 3D imaging mass cytometry 34, enable multi-marker immune cell staining at single-cell resolution, revealing intricate interactions between the ENS and immune system. 35 These insights have the potential to inform novel therapeutic approaches. As AI continues to evolve, integrating 3D imaging with clinical and cohort data will expand its diagnostic applications, setting new standards in fields such as oncology, neuroscience, and immunology.

To fully realize these benefits, future research should focus on refining AI algorithms and employing larger, more diverse datasets to enhance reliability and accuracy. Advanced deep learning models can uncover subtle patterns in complex medical data, offering deeper insights into disease mechanisms. The integration of AI-powered analysis with clinical and cohort datasets will further expand its utility, enabling comprehensive diagnostic frameworks and advancing the development of personalized treatment strategies. This AI-powered 3D imaging approach holds the potential to revolutionize medical diagnostics and treatment by rapidly identifying novel clinical features in a cost-effective manner.

## Supplementary Material

Supplementary legends, methods and figures.

Supplementary video 1.

Supplementary video 2.

Supplementary video 3.

## Abbreviations

2D: Two-Dimensional
3D: Three-Dimensional
AI: Artificial Intelligence
ENS: Enteric Nervous System
ETC: Electrophoretic Tissue Clearing
GI: Gastrointestinal
HSCR: Hirschsprung Disease
IBD: Inflammatory Bowel Disease
IBS: Irritable Bowel Syndrome
IHC: Immunohistochemistry
IRB: Institutional Review Board
MP: Myenteric plexus
PBS: Phosphate-Buffered Saline
PFA: Paraformaldehyde
RI: Refractive Index
SMP: Submucosal plexus
UC: Ulcerative Colitis**.**
