# Supplementary material for: AI-powered 3D pathology protocol enhances enteric nervous system visualization and quantification for clinical diagnostics
Source: Theranostics. 2025 Jun 20;15(15):7440–53. doi: 10.7150/thno.112024 (PMC12315810; doi:10.7150/thno.112024)
Supplement: Supplementary file 1 — Supplementary legends, methods and figures. [file thnov15p7440s1.pdf]

Supplements

Supplementary material 1. Detailed methodology for AI-powered 3D tissue analysis of cleared samples.

Supplementary material 2. Comparative analysis of colonic biopsies from healthy controls (HC) and patients with ulcerative colitis (UC).

Supplementary material 3. Artificial intelligence (AI)-powered three-dimensional reconstruction of ganglionic and aganglionic regions in a Hirschsprung disease patient.

Video S1. Example video on AI image training process.

Video S2. Rotating 3D Visualization of AI-Segmented Colonic Structures in UC and HSCR Samples.

Video S3. A detailed introduction to AI 3D reconstructed images and a comparison between non-AI and AI-powered 3D reconstructed images**.**

Supplementary Material 1. Detailed methodology for artificial intelligence (AI)-powered three-dimensional (3D) tissue analysis of cleared samples.

Initial Workflow for Pre-trained AI Model using 3D Tissue Images

Initial AI Training with Raw 3D Images

- The training process begins by importing a raw 3D tissue image into the AI analysis software.
- The Pixel Classifier function is used to initiate the training.
- Researchers manually annotate two-dimensional (2D) cross-sectional layers by labeling:
    - Positive signals (e.g., target structures such as crypt epithelium or neural fibers)
    - Background signals (e.g., lamina propria, lumen, or artifacts)
- Annotation is performed using mouse-based selection tools within the software.

Iterative “Teach” and “Apply” Cycles

After initial annotations, the software undergoes iterative rounds of supervised learning.

- The software undergoes repeated cycles of:
    - Teaching: where the software learns from annotated signals
    - Applying: where the learned classifier is tested on new layers or samples
- This cycle is repeated across:
    - Multiple 2D layers within the same 3D stack
    - Additional raw image datasets from the same experimental batch
- The goal is to ensure robust and accurate signal detection across varied image conditions.

Pseudo-Channel Generation and Pre-trained Model Export

- Once the classifier successfully distinguishes the target from the background:
    - The recognized features are output as new pseudo-channels
    - These channels represent the segmented structures (e.g., crypt epithelium or neural fibers)
- At this point, the trained parameters are saved as a “recipe” file
    - This file serves as a pre-trained model for future use

Application to New Raw 3D Images

- The saved recipe can now be directly applied to new raw 3D images.
- This eliminates the need for further manual annotation or training.
- The result is consistent 3D rendering and segmentation across similar image datasets.

Application to Biopsy Samples (e.g., Ulcerative colitis)

Preprocessing and Annotation of Biopsy Images

- Raw 3D confocal images of biopsy tissues are first contrast-adjusted at the 2D level.
    - This improves visual distinction of crypts and neural fibers.
- Using the Pixel Classifier:
    - The crypt epithelium is annotated as the target.
    - Lamina propria and crypt lumen are labeled as background.
- Neural fibers are also manually tracked and labeled.

Generation of Segmented Pseudo-Channels

- Iterative teaching/applying cycles generate pseudo-channels for each structure.
- Researchers can verify intermediate outputs before proceeding.
- These pseudo-channels enable visual validation of classifier performance.

Final 3D Reconstruction and Quantification

- A final 3D reconstruction is performed using the pseudo-channels.
- The output is used for quantitative morphometric analysis (e.g., volume, diameter).

Application to Surgical Samples (e.g., Hirschsprung disease)

ROI Selection and Neural Structure Classification

- Confocal images from HSCR patient tissues undergo the same workflow.
- Regions of interest (ROIs) include:
    - Mucosal plexus
    - Submucosal plexus
    - Inner circular muscle
    - Myenteric plexus
    - Outer longitudinal muscle
    - Serosa
- Within each ROI:
    - Neural structures and background are annotated using the Pixel Classifier.
    - Manual “teach/apply” cycles are applied as needed.

Generation of Segmented Pseudo-Channels and Visualization

- Pseudo-channels were generated through iterative training and used to visually verify classifier performance before final rendering.

3D Rendering and Quantitative Analysis

- The trained classifier generates pseudo-channels for each ROI.
- Final 3D reconstructions are rendered for quantitative evaluation of neural features.

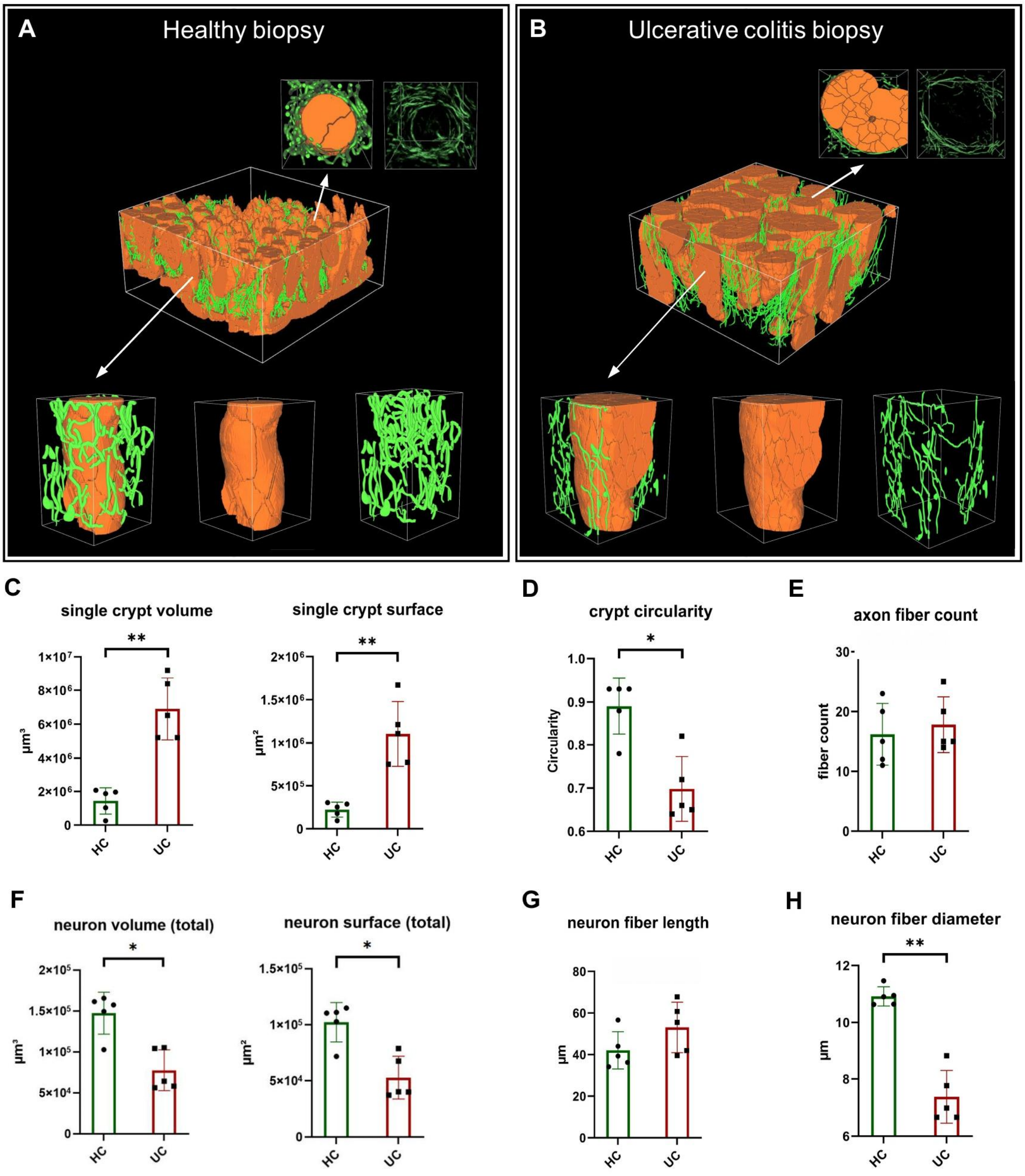

Supplementary Material 2. Comparative analysis of colonic biopsies from healthy controls (HC) and patients with ulcerative colitis (UC). Representative artificial intelligence-generated three-dimensional reconstruction of a (A) healthy colon biopsy sample and (B) a UC colon biopsy sample. (C) Quantification of single crypt volume and surface area revealed significantly increased crypt size in UC compared to HC. (D) Crypt circularity was significantly reduced in UC. (E) The number of axon fibers did not differ significantly between groups. (F) Total enteric neuron volume and surface area were significantly decreased in UC. (G) Neuron fiber length showed no significant difference between groups. (H) Neuron fiber diameter was significantly reduced in UC compared to HC. *$p < 0.05$, **$p < 0.01$

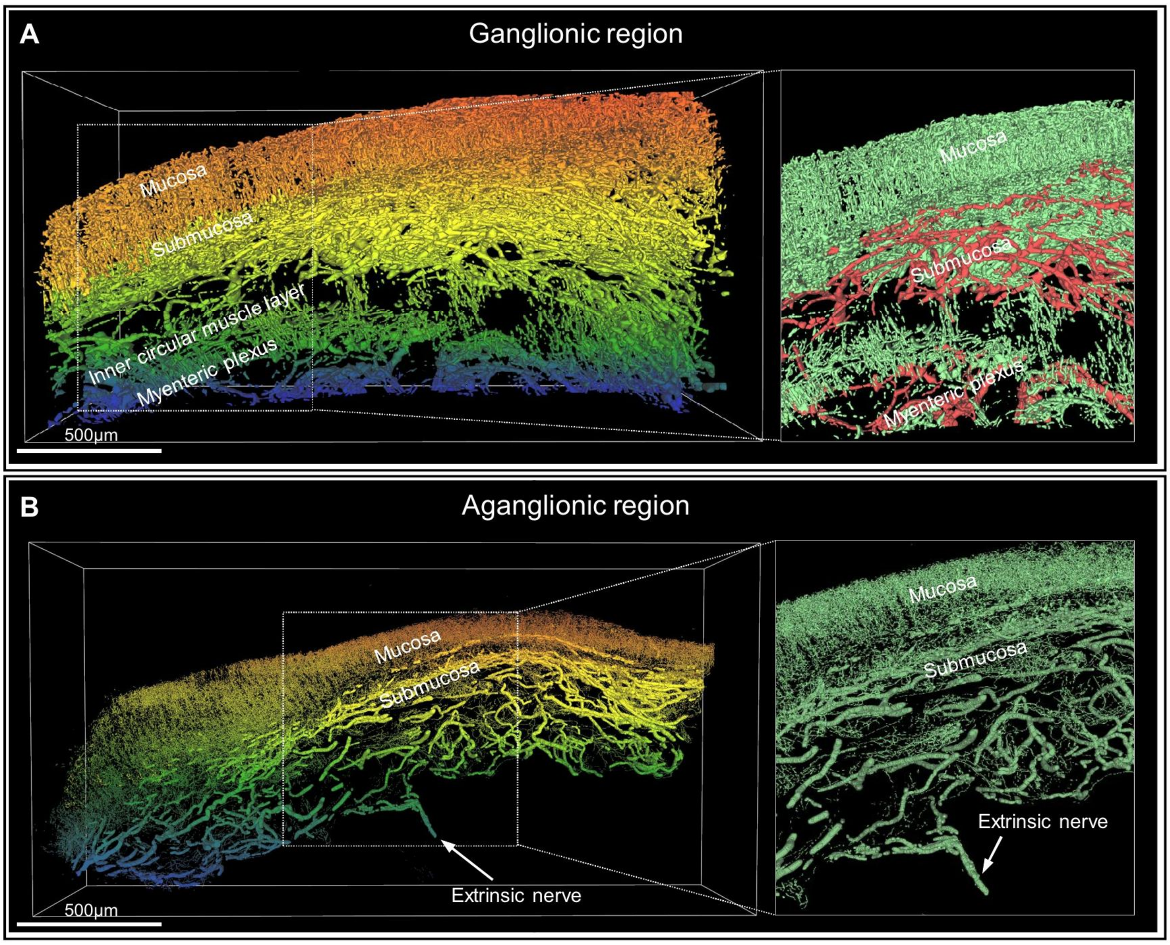

Supplementary Material 3. Artificial intelligence (AI)-powered three-dimensional reconstruction of ganglionic and aganglionic regions in a Hirschsprung disease patient. (A-B) The AI-based segmentation was color-coded by layer, with red representing the mucosal side and blue indicating the serosal side. (A) The ganglionic region contains an intact myenteric plexus (MP) along with other neural structures, whereas (B) the aganglionic region is devoid of the MP and associated neural components.
